# Geometry-Tuned Optical Absorption Spectra of the Coupled Quantum Dot–Double Quantum Ring Structure

**DOI:** 10.3390/nano14161337

**Published:** 2024-08-11

**Authors:** Doina Bejan, Cristina Stan

**Affiliations:** 1 Faculty of Physics, University of Bucharest, 077125 Bucharest, Romania; doinita.bejan@unibuc.ro; 2 Faculty of Applied Sciences, National University of Science and Technology POLITEHNICA Bucharest, 060042 Bucharest, Romania

**Keywords:** coupled quantum dot–double quantum rings, absorption, geometry control, electronic properties

## Abstract

We investigate the energy spectra and optical absorption of a 3D quantum dot–double quantum ring structure of GaAs/Al_0.3_Ga_0.7_As with adjustable geometrical parameters. In the effective mass approximation, we perform 3D numerical computations using as height profile a superposition of three Gaussian functions. Independent variations of height and width of the dot and of the rings and also of the dot–rings distance determine particular responses, useful in practical applications. We consider that a suitable manipulation of the geometrical parameters of this type of quantum coupling offer a variety of responses and, more important, the possibility of a fine adjusting in energy spectra and in the opportunity of choosing definite absorption domains, properties required for the improvement of the performances of optoelectronic devices.

## 1. Introduction

Complex semiconductor nanostructures built using different configurations of quantum dots (QDs), quantum rings (QRs) or a combination of them are considered as artificial molecules having discrete energy spectra and optoelectronic properties that can be easily tuned by carefully modifying their composition, strain, size and shape. They have many potential applications in the construction of terahertz detectors and modulators, efficient solar cells, quantum information technologies, photonic quantum technologies, nanotransistors, etc. [1,2,3,4,5,6,7,8].

For these reasons, the theoretical investigations of QD and QR properties have received much attention in recent years. Since the literature is quite abundant in this direction, we will cite only a few examples from each case. For instance, there are reported theoretical studies about quantum dots coupled laterally [7] or vertically aligned [9,10], about quantum rings laterally or vertically coupled [11,12,13,14] and about vertical stacks of rings [15], double toroidal quantum rings [16], concentric double rings [17,18,19,20,21,22] and triple rings [23,24].

Considerable attention has received the combination of quantum dot with quantum ring. The growth performed with droplet epitaxy enables the combination of QD and QR into a single, multi-functional complex (QDR) [25,26]. The electronic and optical properties of QDRs without or with hydrogenic donor impurity or singly ionized double donor systems (*D*^+2^) were investigated in external electric or magnetic fields [27,28,29,30,31,32,33]. Theoretical studies of the *dc* current through a QDR show that it can efficiently work as a single-electron transistor or a current rectifier [34]. Many particle studies revealed that it is possible to effectively control the electron charge and spin distribution inside QDR by modifying the confinement potentials [35,36,37].

Despite the abundance of studies related to combined dots and rings, to the best of our knowledge, the quantum dot–double ring structure (QDDR) has not yet been theoretically studied even though it was experimentally constructed through droplet epitaxy in the last decade by Somaschini et al. [25]. For this reason, in the present paper we investigate the influence of geometrical parameters on the electronic and optical properties of QDDR. The influence of the external fields on this structure will be the subject of other forthcoming papers.

Because the experimental QDDR has a low response to external fields having a large diameter (about 360 nm), we propose here a smaller structure with adjustable dimensions that we verified as highly responsive to electric and magnetic fields, being in this way the most interesting for potential applications. New generations of electronic and optical devices with a decreasing size are required due to the rapid advancement in communication technology, information systems, nanoelectronics and optoelectronics. We follow the example of Hernandez et al. [32] who, starting from the experimental QDR structure given also in [25,32], investigated a smaller one in order to compare their results with previous ones [38]. Synthesis of QRs or QDs with similar dimensions has been reported by various other authors [39,40,41].

Hernandez et al. [32] also showed that the height profile of the experimental QDR can be successfully described by a superposition of Gaussian functions, which allows the independent control of the QR and QD height-to-base aspect ratios. Similar to the model proposed in [32] as a validation test, the height profile of the quantum dot–double quantum ring was built as a superposition of three Gaussian functions. This theoretical shape allows an independent control for each QR and QD height, width and position, offering the possibility of an in-depth investigation of the effects of geometry variation on the electronic and optical absorption spectra.

We perform full 3D numerical computations that allow obtaining results for the variation of the structure height on a large domain with a higher level of accuracy compared to the usual adiabatic approach for the separation of the in-plane and vertical variables [42]. We also show that the independent variation of each parameter enables the control of the electron density localization in each of the rings or in the quantum dot, a property which is crucial for the transport properties of semiconductor structures [34].

The paper is organized as follows. In Section 2 we describe the theoretical framework for QDDR. The electronic and optical properties are presented and analyzed comparatively in Section 3. Finally, the conclusions are summarized in Section 4.

## 2. Theory

**Figure 1:**
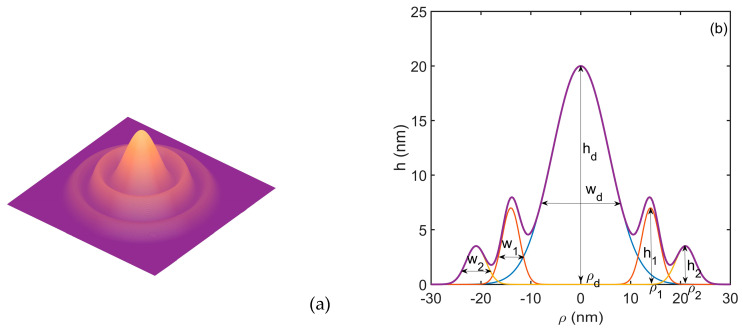
The 3D QDDR structure (**a**) is built by revolving around the *z*-axis the height profile modelled in (**b**) as a superposition of three gaussian functions with specified parameters.

We consider the electrons confined in the coupled quantum dot–double quantum ring of GaAs/Al_0.3_Ga_0.7_As. We use a finite confinement potential *V*(*x*,*y*,*z*) corresponding to the geometry illustrated in Figure 1.

Following [31], the height profile $h\left(\rho \right)$ of the GaAs/AlGaAs structure is constructed as a superposition of three Gaussian functions, each centered on the position of the dot ($\rho_{d}$), inner ring ($\rho_{1}$) and outer ring ($\rho_{2}$):

(1) $$h\left(\rho \right)=h_{d}\exp\left(-\frac{{\left(\rho -\rho_{d}\right)}^{2}}{w_{d}^{2}}\right)+h_{1}\left(-\frac{{\left(\rho -\rho_{1}\right)}^{2}}{w_{1}^{2}}\right)+h_{2}\left(-\frac{{\left(\rho -\rho_{2}\right)}^{2}}{w_{2}^{2}}\right)$$

where $\rho =\sqrt{x^{2}+y^{2}}$, $h_{d},h_{1},h_{2}$ are the maximum height while $w_{d},w_{1},w_{2}$ corresponds to the full width at 1/*e* from the height of the dot, inner ring and outer ring, respectively, as indicated in Figure 1b.

The atemporal Schrödinger equation for the electron in this structure reads as:

(2) $$\left(-\frac{\hbar^{2}}{2}\nabla \left(\frac{1}{m{\left(\vec{r}\right)}^{*}}\nabla \right)+V\left(x,y,z\right)\right)\psi \left(x,y,z\right)=E\psi \left(x,y,z\right)$$

The electron confining potential $V\left(\vec{r}\right)$ and the position-dependent effective mass of the electron $m{\left(\vec{r}\right)}^{*}$ have expressions dictated by the axial symmetry of the structure as follows:

(3) $$\begin{array}{l} V\left(\vec{r}\right)=V_{0}\left(H\left(-z\right)+H\left(z-h\left(\rho \right)\right)\right)\\ m{\left(\vec{r}\right)}^{*}={m^{*}}_{GaAs}+\left({m^{*}}_{GaAlAs}-{m^{*}}_{GaAs}\right)\left(H\left(-z\right)+H\left(z-h\left(\rho \right)\right)\right) \end{array}$$

Here *H* is the Heaviside step function and $V_{0}$ is the barrier potential for electrons in GaAs embedded in Ga_0.7_Al_0.3_As.

The energy eigenvalues *E* and eigenfunctions $\psi \left(x,y,z\right)$ were calculated numerically using FEM (finite element method) as incorporated by COMSOL Multiphysics^®^ software (v. 5.6) [43]. The spatial domain of integration of the model has a cylindrical shape, coaxial with the QDDR, having a radius and a height twice the corresponding dimensions of the structure. We used an adaptative, free tetrahedral-type mesh and Dirichlet conditions for the boundary of the cylindrical domain.

When the QDDR system is under the action of a probe laser of variable angular frequency (ω), the intraband absorption coefficient for transitions starting from the ground level can be written, using the compact density-matrix formalism under the steady state conditions, as [44]:

(4) $$\alpha_{1j}\left(\omega \right)=\frac{\omega N\mu_{1j}^{2}T_{2}}{\varepsilon_{0}\hbar cn_{r}}\frac{\left|J_{0}^{2}\left(\frac{\left|\mu_{jj}-\mu_{11}\right|E_{0}}{\hbar \omega }\right)-J_{2}^{2}\left(\frac{\left|\mu_{jj}-\mu_{11}\right|E_{0}}{\hbar \omega }\right)\right|}{1+T_{2}^{2}{\left(\omega -\omega_{j1}\right)}^{2}+{\bar{\mu }}_{1j}^{2}E_{0}^{2}T_{1}T_{2}/\hbar^{2}}$$

where

(5) $${\bar{\mu }}_{1j}=\mu_{1j}\left(J_{0}\left(\frac{\left|\mu_{jj}-\mu_{11}\right|E_{0}}{\hbar \omega }\right)+J_{2}\left(\frac{\left|\mu_{jj}-\mu_{11}\right|E_{0}}{\hbar \omega }\right)\right)$$

In Equations (4) and (5), $J_{0},J_{2}$ are the first-kind Bessel functions of orders 0 and 2, N is the electron density, T_1_ is the population decay time and T_2_ is the dephasing time, $\varepsilon_{0}$ is the vacuum dielectric permittivity, *n_r_* is the refractive index and *c* the vacuum speed of light. $E_{0}$ is the amplitude of the probe laser electric field, and $\mu_{ij}$ are the dipole moment matrix elements between the states *i* and *j*. However, because of the axial symmetry, all $\mu_{jj}$ are negligibly small, so the part related to the Bessel functions is always 1.

## 3. Results

In this section, we present the numerical results concerning the geometry effects on the electronic and optical properties of the QDDR structure. The parameters used in our computations are: ${m^{*}}_{GaAs}=0.067\cdot m_{0}$, ${m^{*}}_{GaAlAs}=0.093\cdot m_{0}$ (where $m_{0}$ is the mass of a free electron), $V_{0}$ = 262 meV [45], $n_{r}=3.55$, *T*_1_ = 10 ps, *T*_2_ = 5 ps [44], $N=5\times 10^{22}\;m^{-3}$.

If not specified otherwise, the QDDR parameters are: $h_{d}$ = 20 nm, $w_{d}$ = 8 nm, $\rho_{d}$ = 0 for the dot (QD), $h_{1}$ = 7 nm, $w_{1}$ = 2.5 nm, $\rho_{1}$ = 14 nm for the inner ring (QR1) and $h_{2}$ = 3.5 nm, $w_{2}$ = 2.8 nm, $\rho_{2}$ = 21 nm for the outer ring (QR2). The parameters for the dot and inner ring are taken from [32] to facilitate the comparison of the results. The values for the outer ring parameters are chosen proportional to those of the inner ring, their ratios being the same as for the experimental structure from [25]. We varied each parameter independently in order to observe its specific influence on the energy spectra and absorption coefficient. We consider that ten levels of energy are relevant for the electronic and absorption properties in all the considered cases. In the following, only the most representative results are presented. We mention that, in all calculations, the QDDR structure keeps always its cylindrical symmetry.

### 3.1. Electronic Properties of the Quantum Dot–Double Quantum Ring

**Figure 2:**
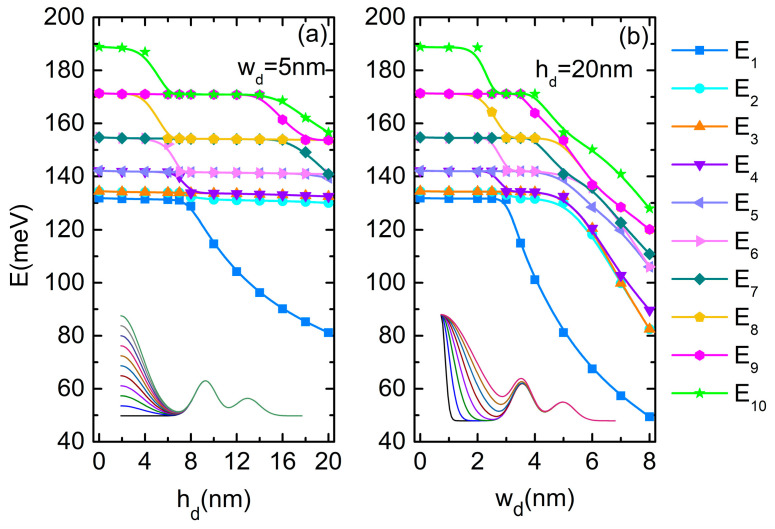
The energies of the ten lowest states of the electron in DDQR: (**a**) as functions of *h_d_*; (**b**) as functions of *w_d_*. The insets represent the variation of the height profile on the positive values of ρ.

We analyze first the influence of the central dot parameters. Figure 2a,b present the variation of the electron energies in QDDR as functions of the dot height $h_{d}$ (at $w_{d}$ = 5 nm) and as a function of *w_d_* (for *h_d_* = 20 nm), respectively. Inset of each figure are the shapes of the semi-profiles for the chosen parameters $h_{d}$ and $w_{d}$, respectively, of the central dot. Additional simulations showed that for other choices of *w_d_*, the energy variation is less affected.

The spectra from Figure 2a,b show multiple anti-crossings due to the lowering of the energy of the state confined into the dot at the increment of $h_{d}$ and $w_{d}$, respectively. Similar anti-crossing in the energy spectra were obtained before by Hernandez et al. [32] for the dot–single ring structure.

**Figure 3:**
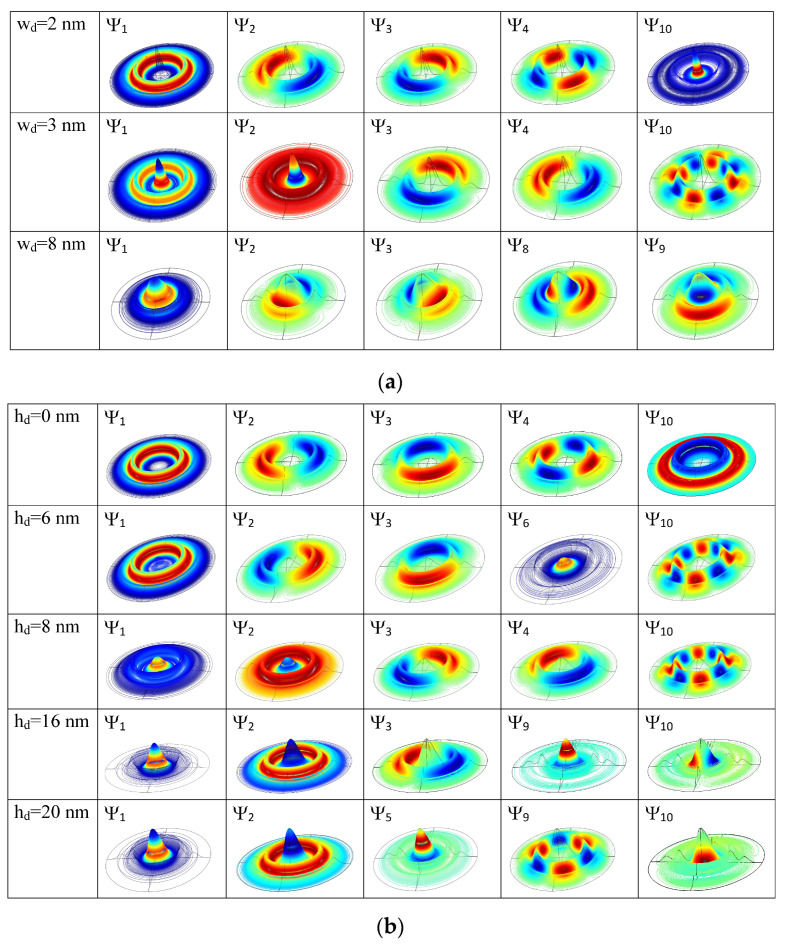
The 3D representation of the QDDR wave functions ordered horizontally in ascending order of energies: (**a**) at different values of *h_d_* for *w_d_* = 5 nm and (**b**) at different values of *w_d_* for *h_d_* = 20 nm. At each *h_d_* and *w_d_* values are indicated the corresponding wave functions.

An essential support in understanding the energy behavior is given by the 3D representations of the wave functions shown in Figure 3a,b using 500 contour lines.

A limiting case is the quantum dot absence ($h_{d}$ = 0), when the structure corresponds to a double concentric quantum ring (DQR). The ground level is single, with the quantum magnetic number *m* = 0, and, due to the cylindrical symmetry, all the other levels form degenerate pairs (with quantum magnetic numbers *m*, −*m*) [45]. As shown in Figure 3a, first row, the wave function (WF) of the ground state ψ_1_ has a maximum located on the inner ring and a minimum on the outer one because of the greater height of the inner ring. Its energy is E_1_ = 131.8 meV. The WFs of the second and third states (with *m* = 1, −1) have one maximum and one minimum, both located on the inner ring. The fourth and fifth states’ WFs (with *m* = 2, −2) have two maxima and two minima, all located on the inner ring, and so on. However, the tenth state is a single state, completely symmetrical (*m* = 0) but, contrary to ψ_1_, with a minimum located on the inner ring and a maximum on the outer ring.

As the height or width of the central quantum dot grows, the effects in the energy levels (Figure 2) and wave function localizations (Figure 3) start to be significant after some critical values. The WFs illustrated in the first column in Figure 3 show the effect of the increasing of the quantum dot height or width on the ground state WF and, implicitly, on the probability of the electron localization in QDDR.

For small values of $h_{d}$, the WF of the ground state keeps its maximum on the inner ring. At $h_{d}$ = 6 nm, the energies of the excited levels decrease abruptly and E_6_ is now the single level with a WF maximum into the dot (second row of Figure 3a). New degenerate pairs of levels are formed (E_9_, E_10_), (E_8_, E_7_) and, due to their re-grouping, multiple anti-crossing points appear in the energy spectrum of Figure 2a. At $h_{d}$ = 8 nm (third row of Figure 3a), ψ_1_ and ψ_2_ completely change their configuration: ψ_1_ has a maximum within the dot and a minimum on the DQR, while for ψ_2_ the locations of maxima and minima are reversed. The formation of new pairs (E_3_, E_4_), (E_5_, E_6_) determines other anti-crossing in the spectrum. At further increment of $h_{d}$, the WF of the ground state becomes more and more confined into the dot and its energy decreases as a direct consequence of $h_{d}$ raise. Up to $h_{d}$ = 14 nm, the degenerate pairs maintain a constant energy, because their WFs are confined in the DQR. At $h_{d}$ = 16 nm, ψ_2_–ψ_8_ are eigenfunctions with maxima and minima located mainly on QR1. ψ_9_ and ψ_10_ present maxima and minima mainly within the dot (fourth row of Figure 3a), and the corresponding states separate their energies because, as expected, (E_10_, E_11_) becomes degenerate. Finally, at $h_{d}$ = 20 nm, ψ_2_–ψ_4_ and ψ_6_–ψ_9_ have maxima and minima located mainly on QR1, ψ_5_ is confined mainly into the dot (as the third single state, completely symmetrical) as well as ψ_10_, leading to new anti-crossings in the energy spectrum (last row of Figure 3a).

As seen in Figure 2b, the energies are more sensitive in their decreasing tendency to the dot width variation, so their control and/or adjustment can be chosen accordingly. The multiple anti-crossing points in the energy spectrum are explained by the WF localization illustrated in Figure 3b. For instance, at $w_{d}$ = 2 nm, all the lowest nine states are confined in DQR, while ψ_10_ has a maximum within the dot and minima on DQR (first row of Figure 3b). The first two states change at $w_{d}$ = 3 nm, extending over the whole QDDR (second row of Figure 3b), while the others are confined in DQR, leading to a re-grouping of degenerate levels and multiple anti-crossings in the energy spectrum. At the further increment of $w_{d}$, due to the dot extension over the inner ring, the ground state energy decreases since the WFs are confined more and more into the dot and QR1. Finally, at $w_{d}$ = 8 nm, ψ_1_–ψ_7_ and also ψ_10_ are all confined into the dot and QR1, while ψ_8_–ψ_9_ extend over QR2 (last row of Figure 3b).

In the second step of our analysis, we are interested in the inner ring influence on the energy spectrum. All the calculations are performed for $h_{d}$ = 8 nm and $w_{d}$ = 8 nm, at which a significant variation of the ground state energy is obtained. Our computations show that at higher values of $h_{d}$, low or insignificant variation can be observed for the ground state energy, similarly to [32].

**Figure 4:**
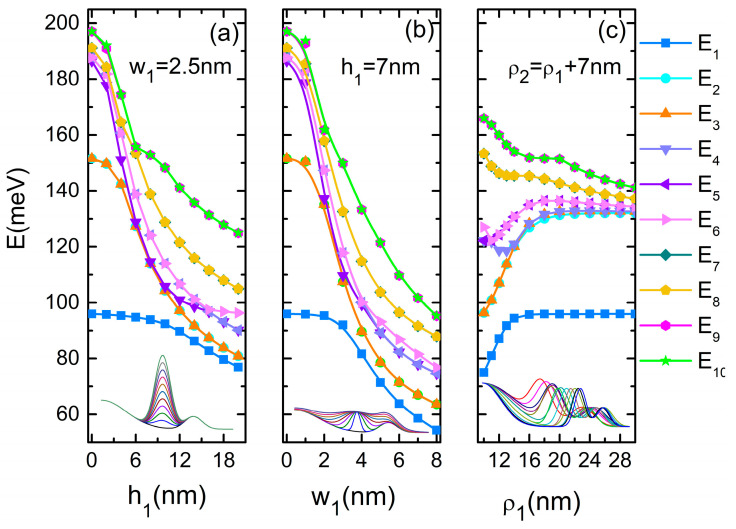
The energies of the ten lowest states of the electron in QDDR: (**a**) as functions of *h*_1_; (**b**) as functions of *w*_1_; (**c**) as functions of *ρ*_1_. The insets represent the variation of the height profile on the positive values of ρ.

**Figure 5:**
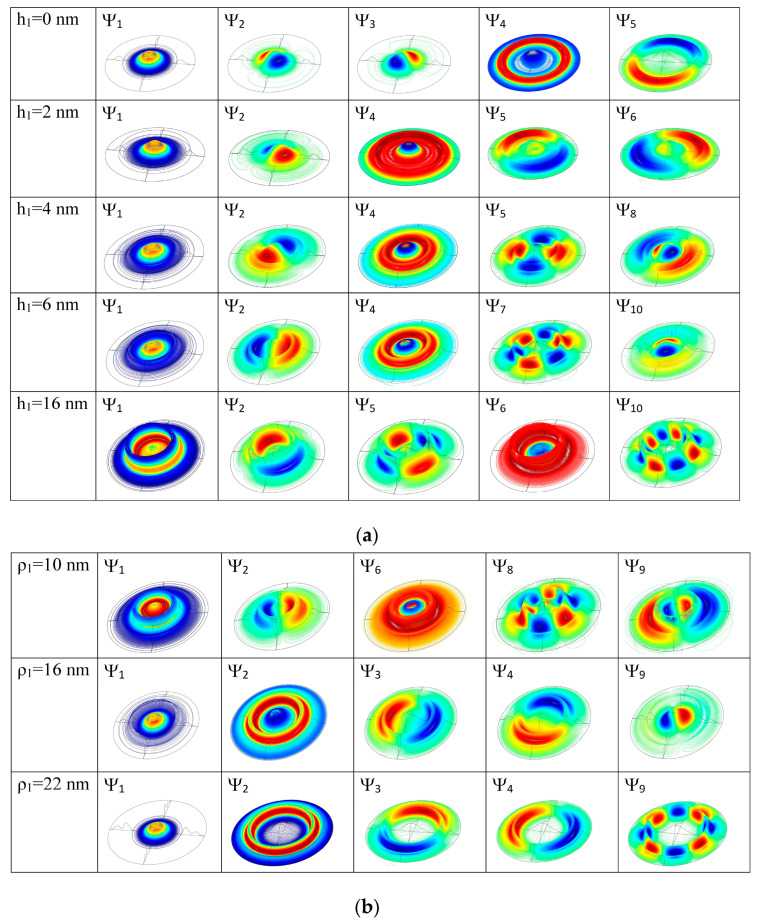
The 3D representation of the QDDR wave functions ordered horizontally in ascending order of energies: (**a**) at different values of *h*_1_ for *w*_1_ = 2.5 nm; (**b**) at different values of *ρ*_1_ for *h*_1_ = 7 nm, *w*_1_ = 2.5 nm and *ρ*_2_ = *ρ*_1_ + 7 nm. At each *h*_1_ and *ρ*_1_ value, the corresponding wave functions are indicated.

Figure 4 presents the electron energies’ response to the variation of the geometric dimensions of QR1. This is discussed in correspondence to the WFs from Figure 5. For the limiting situation $h_{1}$ = 0, the structure corresponds to a quantum dot coupled with the outer ring (Figure 4a). As seen in Figure 5a, first row, the lowest three states are mainly the dot states while, beginning with the fourth level, the upper states are mainly QR2 states. The ground level is single and coincides with the ground level of the QD (E_1_ = 95.93 meV). (E_2_, E_3_) form a degenerate pair and E_4_ is a single state with a different radial function than ψ_1_, while (E_5_, E_6_), (E_7_, E_8_), (E_9_, E_10_) form new degenerated pairs. For $h_{1}$ = 2 nm (Figure 5a, second row), the first three states are mainly located in QD, but the upper ones extend over QDDR, because the presence of QR1 facilitates the coupling between the QR2 and the dot. When $h_{1}$ = 4 nm (Figure 5a, third row) the lowest three states extend over the dot and inner ring and the fourth state over the whole QDDR with a maximum on the inner ring (single state). WFs ψ_7_ and ψ_8_ have *m* = 1, −1 like ψ_2_, ψ_3_ but different radial function. At $h_{1}$ = 6 nm, due to the anti-crossing between the degenerate levels (E_7_, E_8_) and (E_9_, E_10_), ψ_9_, ψ_10_ have now *m* = 1, −1.

At the further increment of $h_{1}$, the lowest three states cover more and more of the whole structure. There is a clear transition (around 10 nm) to a linear decrement in the ground state energy. This is generated by the spreading of this state over the whole QDDR, indicating a lowering of the confinement. Around $h_{1}$ = 16 nm, E_6_ becomes the single level due to the anti-crossing between E_4_ and the degenerate levels (E_5_, E_6_). Moreover, the WFs ψ_1_–ψ_6_ extend the whole structure while ψ_7_–ψ_10_ are confined mainly into the DQR (last row in Figure 5a).

Figure 4b illustrates the variation of the electron energies as functions of $w_{1}$. We can observe a great similarity with Figure 4a (for this reason, we do not represent the WFs), except that the structure is more sensitive to $w_{1}$ increment. For instance, the passage to a linear decrement in the ground state energy is observed for $w_{1}$ > 3 nm.

Figure 4c presents the variation of the electron energies as functions of $\rho_{1}$. We modified $\rho_{1}$ from 10 nm to 30 nm, maintaining a constant distance between QR1 and QR2 ($\rho_{2}=\rho_{1}+7\;\mathrm{nm}$). Therefore, we modified only the distance between QD and DQR.

We observe that the energies of the lowest three states increase and then stabilize at constant values as $\rho_{1}$ grows. On the contrary, the energies of the upper states decrease, first rapidly and then more slowly. This behavior can be explained in connection with the selected WFs illustrated in Figure 5b.

At $\rho_{1}$ = 10 nm, ψ_1_–ψ_5_ extend over the whole QDDR, while ψ_6_–ψ_8_ are confined mainly in DQR (with very little extension in the dot) and ψ_9_–ψ_10_ completely cover the dot. E_1_ and E_6_ are single states while all other levels form degenerate pairs (Figure 5b, first row). Beginning with $\rho_{1}$ = 16 nm, ψ_1_, ψ_9_, ψ_10_ are almost confined into the dot, while ψ_2_–ψ_8_ are located in DQR with a little extension into the QD region (Figure 5b, second row). Thus, E_1_, E_2_ are single levels, all other levels forming degenerate pairs. At the further increment of $\rho_{1}$, E_1_ and E_2_ stabilize at constant values of 95.9 meV and 131.9 meV, respectively, because they are the ground states of the QD and DQR. Beginning with $\rho_{1}$ = 22 nm, all excited states are WFs of DQR due to the increasing distance between QD and DQR (Figure 5b, last row). However, the energies of the upper levels still decrease as their WFs are restrained more and more in the DQR region.

In the third step of our analysis, we are interested in observing the effects of the parameters related to the second ring on the energy spectra.

**Figure 6:**
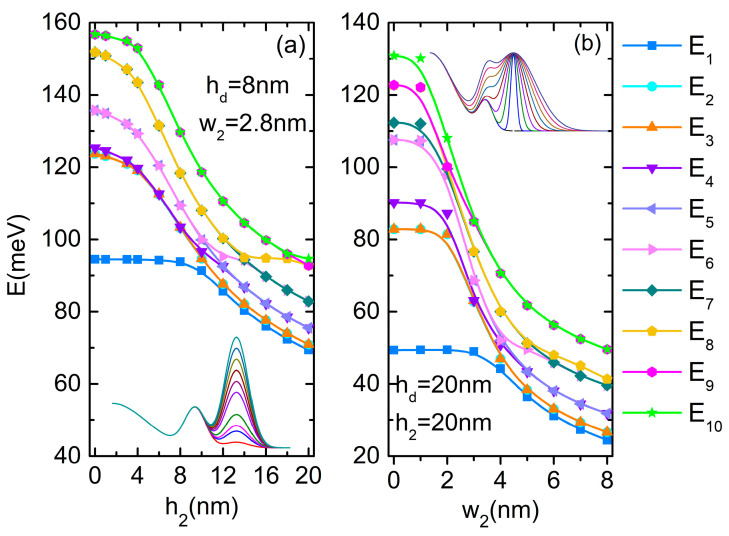
The energies of the ten lowest states of the electron in QDDR: (**a**) as functions of *h*_2_, $w_{d}$ = 8 nm; (**b**) as functions of *w*_2_. The insets represent the variation of the height profile on the positive values of ρ.

**Figure 7:**
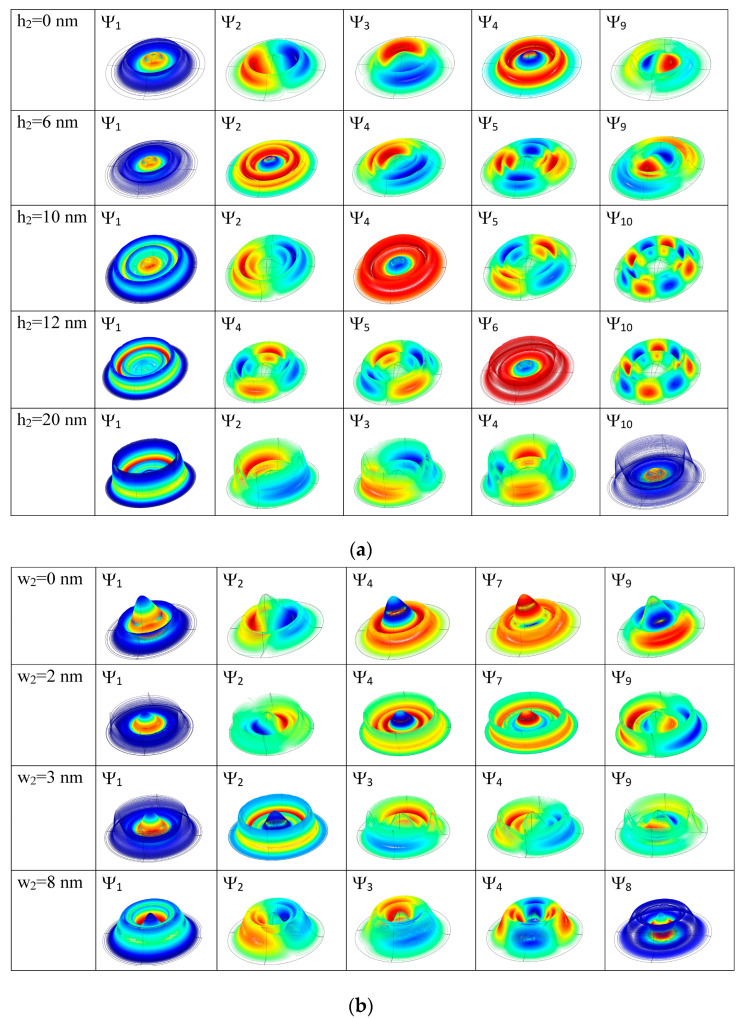
The 3D representation of the QDDR wave functions ordered horizontally in ascending order of energies: (**a**) at different values of *h*_2_ for *w*_2_ = 2.8 nm; (**b**) at different values of *w*_2_ for *h*_2_ = 20 nm. At each *h*_2_ and *w*_2_ value, the corresponding wave functions are indicated.

Figure 6a presents the variation of the electron energies as functions of $h_{2}$ at $w_{2}$ = 2.8 nm and $h_{d}$ = 8 nm. As above, it is discussed in correspondence to WFs from Figure 7a. At $h_{2}$ = 0, the structure is a dot coupled with one ring (QR1). The ground level has the energy 94.48 meV, close to the ground state energy of the dot (95.93 meV). The fourth state is single, with the WF minim on QD and maxim on QR1. Excepting E_1_ and E_4_, all other levels come into pairs. At $h_{2}$ = 6 nm, the energy of the ground state remains constant because its WF is still maxim on the QD. The second state becomes single (Figure 7a, second row). At $h_{2}$ = 10 nm, there is a decrease in the energy of the ground state because its WF spreads over the whole structure. The single state is ψ_4_ leading to new anti-crossings that propagate in the energy spectrum at *h*_2_ increment, because of the rather constant energy of this single state (since around 94.5 meV is confined mainly in the QD—Figure 7a, third row), while all other energies decrease. For instance, at $h_{2}$ = 12 nm, the single state is the sixth (Figure 7a, fourth row), and for $h_{2}$ = 14–18 nm it becomes the eighth state. Finally, at $h_{2}$ = 20 nm, ψ_10_ is confined mainly into the QD, with energy close to the QD ground state, opposite to the lowest nine states, which are confined mainly into the DQR (Figure 7a, last row).

Figure 6b shows the modification of electron energies with $w_{2}$ variation for $h_{2}$ = $h_{d}$ = 20 nm. We observe similarities with Figure 6a especially in the decreasing tendency, even if the dot height is different. The ground state energy for $w_{2}$ = 0 is 49.34 meV, lower than for the QD ground energy (51.09 meV for $w_{d}$ = 8 nm, $h_{d}$ = 20 nm) because of the WF extension over QR1 (Figure 7a, first row). The second single state with a WF minim on QD and maxim on QR1 is ψ_4_. The upper levels are not all paired because of the appearance of a third single level, E_7_, a completely symmetrical state with WF maxima on both QD and QR1. At $w_{2}$ = 2 nm, E_7_ comes very close to (E_8_, E_9_) but it is not degenerate with them, as can be seen in Figure 7b, second row. Afterward, the energy of this third single state decreases more slowly than E_8_–E_10_, going out from the analyzed levels. At $w_{2}$ = 3 nm, the second state becomes single (Figure 7b, third row) and, as seen from Figure 6b, its energy decreases quickly up to $w_{2}$ = 4 nm, then slowly reduces at the further increment of $w_{2}$. At $w_{2}$ = 8 nm, the ground state extends over the whole QDDR, while ψ_8_ is the second single state, confined mainly in the QD (Figure 7b, last row).

### 3.2. Optical Properties of the Triple Concentric Quantum Rings

We consider only the transitions starting from the ground state, induced by a probe laser of intensity 5 × 10^7^ W/m^2^. We discuss the nonlinear optical absorption of QDDR in connection with the oscillator strength that is defined as

(6) $$O_{1-j}=\frac{2{m^{*}}_{GaAs}}{\hbar }\Delta E_{1j}\mu_{1j}^{2}=\frac{2{m^{*}}_{GaAs}}{\hbar }\Delta E_{1j}e^{2}{\left(∭\psi_{1}x\psi_{j}dxdydz\right)}^{2}$$

for the transitions induced by an *x*-polarized probe laser. Each absorption coefficient is the sum over $\alpha_{1j}$, *j* = 2–10. The considered transition energies are below 95 meV.

**Figure 8:**
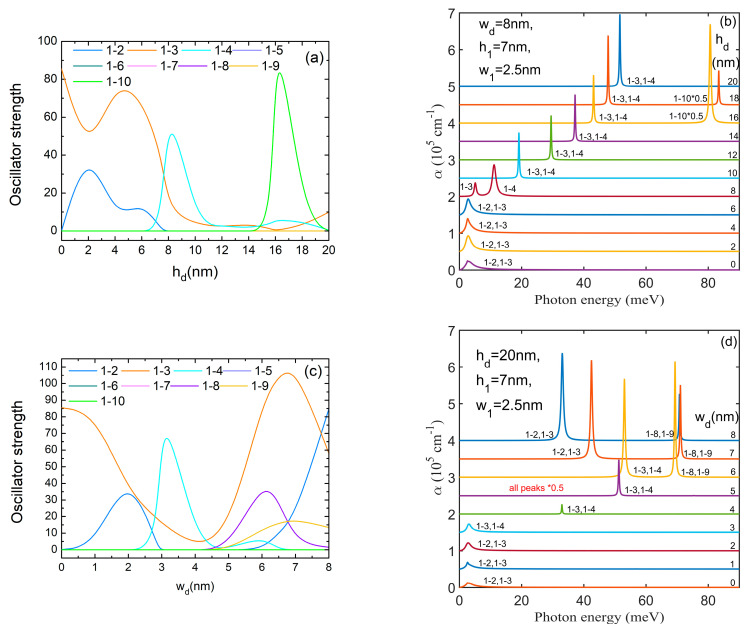
(**a**) The oscillator strengths as functions of *h_d_*; (**b**) the absorption spectra at different values of *h_d_*; (**c**) the oscillator strengths as functions of *w_d_*; (**d**) the absorption spectra at different values of *w_d_*. The transitions are indexed as 1–*j*, from the ground level to the excited *j* level. To avoid spectra overlapping, each spectrum is translated on the vertical axis.

Figure 8a,c present the oscillator strength behavior at $h_{d}$ and $w_{d}$ variations, respectively. In both graphs, we notice that *O*_1–3_ takes considerable values on large regions of $h_{d}$ (from 0 to 7 nm) and $w_{d}$ (0–2 nm and 5–8 nm) because ψ_1_ is completely symmetric relative to the *x*, *y* axes, while ψ_3_ does not have a specific symmetry relative to them. Another non-zero oscillator strength is *O*_1–2_ at low values of $h_{d}$ and $w_{d}$ (see Figure 3). At higher values of $h_{d}$ and for $w_{d}$ = 3–6 nm, ψ_2_ becomes completely symmetric relative to the *x*, *y* axes, thus *O*_1–2_ = 0 and *O*_1–4_ ≠ 0 as (E_3_, E_4_) form a pair of non-degenerate levels with ψ_3_, ψ_4_ having *m* = ±1 (see Figure 2 and Figure 3). At higher values of $w_{d}$, ψ_2_ again changes its symmetry (Figure 3b) and *O*_1–2_ grows again while *O*_1–4_ = 0. For $h_{d}=$16 nm, *O*_1–3_ = 0 because ψ_3_ is antisymmetric to the *x*, *y* axes (Figure 3a, fourth row). Also, for $h_{d}=$16–18 nm, *O*_1–10_ takes high values because ψ_10_ is mainly confined into the QD and does not have a specific symmetry relative to the *x*, *y* axes. For $w_{d}=$5–8 nm, *O*_1–8_ and *O*_1–9_ take appreciable values because of the good superposition of ψ_1_ over ψ_8_, ψ_9_ (their WFs also have *m* = ±1, as can be seen in Figure 3b, last row).

Therefore, in the absorption spectra represented in Figure 8b,d, we notice at low $h_{d}$ and $w_{d}$ only the peaks 1 → 2 and 1 → 3. Due to the degeneracy of (E_2_, E_3_) levels, they appear as a single peak. Even if the oscillator strengths are large for these transitions, the corresponding peaks are large but not intense, due to the small transition energies. In both figures, these peaks appear at almost constant transition energies, corresponding to rather constant values of E_1_ and (E_2_, E_3_) in Figure 2. Beginning with $h_{d}$ = 8 nm ($w_{d}=$ 3–6 nm), the peak 1 → 2, 1 → 3 is replaced by 1 → 3, 1 → 4, which has higher energies and a growing intensity with increasing $h_{d}$ (or $w_{d}$). This is due to the rapid lowering of the ground state energy while E_3_, E_4_ remain constant (Figure 2). The large values of *O*_1–10_ in Figure 8a lead to a rather intense absorption peak of 5.4 × 10^5^ cm^−1^ for $h_{d}$ = 16 nm (reduced 0.5 times in Figure 8b).

**Figure 9:**
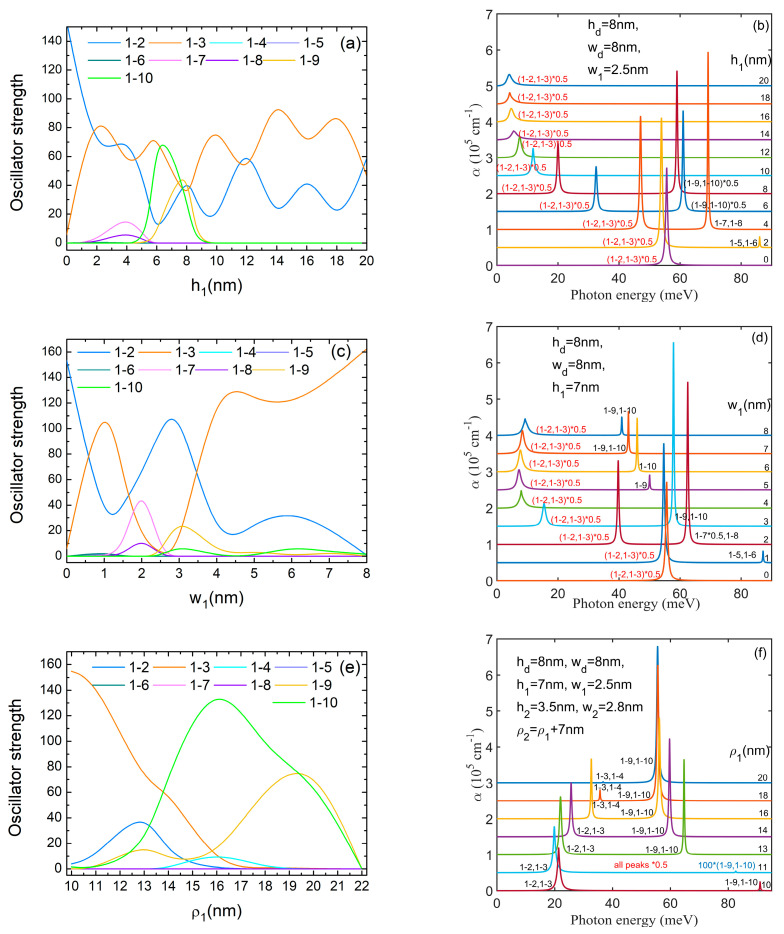
(**a**) The oscillator strengths as functions of *h*_1_; (**b**) the absorption spectra at different values of *h*_1_; (**c**) the oscillator strengths as functions of *w*_1_; (**d**) the absorption spectra at different values of *w*_1_; (**e**) the oscillator strengths as functions of ρ_1_; (**f**) the absorption spectra at different values of *ρ*_1_. The transitions are indexed as 1–*j*, from the ground level to the excited *j* level. To avoid spectra overlapping, each spectrum is translated on the vertical axis.

Figure 9a,c,e present the oscillator strength behavior with $h_{1},w_{1}$ and $\rho_{1}$ variations, respectively. Figure 9a,c are dominated by the large values of *O*_1–2_ and *O*_1–3_, which have an oscillatory and mirror behavior of each other due to fact that the maxima and minima of ψ_2_ and ψ_3_ are perpendicularly oriented and make different angles with the *x*, *y* axes with increasing $h_{1},w_{1}$. On small domains, *O*_1–7_, *O*_1–8_ and *O*_1–9_, *O*_1–10_ take considerable values. For instance, for $h_{1}$ = 4 nm, ψ_7_, ψ_8_ form a pair of functions with *m* = ±1, as can be seen in Figure 5a, third row.

On the other hand, in Figure 9e we notice significant values of *O*_1–9_, *O*_1–10_ for all values of $\rho_{1}$ while *O*_1–2_, *O*_1–3_ are important only for small values. For instance, in Figure 5b, third row, at $\rho_{1}$ = 16 nm, ψ_1_, ψ_9_, ψ_10_ are mainly confined into the QD and ψ_2_–ψ_8_ are mainly confined in DQR. Moreover, ψ_2_ is a symmetric function in *x*, *y*, so only *O*_1–3_, *O*_1–4_ take small values. Beginning with $\rho_{1}$ = 22 nm, all oscillator strengths are zero, because there is no superposition between the ground state confined in the dot and the excited states confined in DQR.

In the absorption spectra shown in Figure 9b,d, a displacement of all peaks toward lower energy with increasing $h_{1},w_{1}$ can be observed. Even if the ground state energy is almost constant at low $h_{1},w_{1}$ and decreases at their increment, similar to its behaviour with increasing $h_{d},w_{d}$, the transition energy of the 1 → 2, 1 → 3 peak diminishes at low $h_{1},w_{1}$ and stabilizes further because of the decrement of the excited states energies. This is in contrast to these peaks’ behaviour at $h_{d},w_{d}$ increment. The large values of *O*_1–2_ and *O*_1–3_ traduce in large peaks at great values of $h_{1},w_{1}$ where they involve small transition energies and in intense ones at small values of $h_{1},w_{1}$ where the transition energies are larger. The intensity is so great that the 1 → 2, 1 → 3 peak is reduced by 0.5 times. The maximum value of 7.28 × 10^5^ cm^−1^ is obtained for this absorption peak at $h_{1}$ = 2 nm. Values of little difference were obtained for the 1 → 9, 1 → 10 peak at $h_{1}$ = 8 nm and the 1 → 2, 1 → 3 peak at $h_{1}$ = 4 nm. Also, comparable values are found for the 1 → 2, 1 → 3 peak at $w_{1}$ = 1 nm and the 1 → 7, 1 → 8 peak at $w_{1}$ = 2 nm.

In the absorption spectra from Figure 9f, we notice large maxima for the 1 → 9, 1 → 10 peak that displaces to lower energies at $\rho_{1}$ increment, and smaller absorption maxima for the 1 → 2, 1 → 3 peak that moves to higher energies. The largest absorption maximum of about 7.5 × 10^5^ cm^−1^ was obtained for the 1 → 9, 1 → 10 peak at $\rho_{1}$ = 18 and 20 nm.

**Figure 10:**
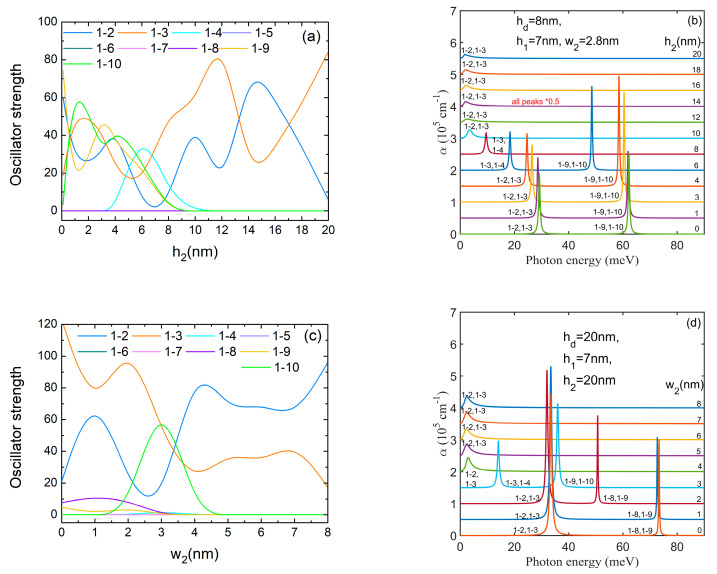
(**a**) The oscillator strengths as functions of *h*_2_; (**b**) the absorption spectra at different values of *h*_2_; (**c**) the oscillator strengths as functions of *w*_2_; (**d**) the absorption spectra at different values of *w*_2_. The transitions are indexed as 1–*j*, from the ground level to the excited *j* level. To avoid spectra overlapping, each spectrum is translated on the vertical axis.

Figure 10a,c show the oscillator strength behavior at $h_{2},w_{2}$ variations, respectively. For similar reasons, we notice again the oscillatory and mirror behavior of *O*_1–2_ and *O*_1–3_. At low values of $h_{2}$, *O*_1–9_, *O*_1–10_ take considerable values while at low values of $w_{2}$, *O*_1–8_, *O*_1–9_ are non-zero because either ψ_9_, ψ_10_ or ψ_8_, ψ_9_ have WFs with *m* = ±1, as can be seen in Figure 7. For instance, at $h_{2}$ = 10 nm, the WFs ψ_5_–ψ_10_ are confined mainly in DQR while ψ_4_ is a symmetric state, so beginning with this value of $h_{2}$ only *O*_1–2_, *O*_1–3_ ≠ 0 (see Figure 7a, third and fourth rows). A similar behaviour was found for $w_{2}\geq$ 4 nm.

In the corresponding absorption spectra we observe the displacement of all peaks toward lower energy with increasing $h_{2},w_{2}$ similar to the spectra obtained at $h_{1},w_{1}$ increment. Beginning with $h_{2}$ = 10 nm ($w_{2}=$ 4 nm) the spectrum is represented only by the 1 → 2, 1 → 3 peak placed at very low energy (2–3 meV). The largest absorption maximum of about 6.9 × 10^5^ cm^−1^ was obtained for the 1 → 9, 1 → 10 peak at $h_{2}$ = 3–4 nm.

From the analysis of the absorption spectra presented in Figure 8, Figure 9 and Figure 10, we can say that, either at low values of $h_{d}$ or $w_{d}$ or at large values of $h_{1}$, $h_{2}$ or $w_{2}$, the spectra are composed of a single peak of low energy. Otherwise, the spectra are composed of two peaks well separated in energy, some of them of rather high intensity. The increment of QDDR parameters is able to induce a blue-shift of all absorption peaks ($h_{d}$), a red-shift of all peaks ($h_{1}$, $w_{1}$, $h_{2},\;w_{2}$) or a blue-shift of the 1 → 2, 1 → 3 peak and a red-shift of the 1 → 9, 1 → 10 peak ($\rho_{1}$). This tunability of the absorption spectra can suggest new opportunities in designing new optical devices based on these structures.

## 4. Conclusions

We have studied the effects of geometry variation on the energy spectra and optical absorption of quantum dot–double quantum ring. We performed 3D numerical computations using a potential build from the cross-sectional profile inferred from a real dot–double ring structure [24].

The profile is mathematically modeled as a superposition of three Gaussian functions, each centered on the position of the dot (QD), inner ring (QR1) and outer ring (QR2). We varied seven independent parameters related to the dot and rings height, width and position and present the most relevant results of our computations.

In the effective mass approximation, we performed a ten-level analysis of the energy, oscillatory strength and absorption spectra. Keeping comparable values for the height of the dot and inner ring, we managed to obtain a significant variation of the ground state energy depending on the rings parameters.

For the physical explanation of the QDDR response to the geometrical constraints, we have used the important support given by the 3D representation of the electron wave functions.

Our results reveal that the energies of QDDR are more sensitive to the width $w_{i}$ variation where *i* = *d*, 1, 2. At the increment of each $h_{i},w_{i}$, the energy of the ground state is rather constant for small values but decreases almost linearly for higher values due to the lowering of the confinement. However, the energies of the excited states show a different behavior. At the increment of quantum dot height ($h_{d}$), the excited state energies are rather constant with small punctual variations, while at the increment of its width ($w_{d}$), the excited state energies are constant up to a specific value and then decrease. The energy spectra present many anti-crossings due to the lowering of the energy of the state confined into the dot. On the other hand, in the case of manipulation of the QR geometry, the energies of the excited states only decrease with the increment of their height and width. The anti-crossings are now determined by the appearance of a second completely symmetrical state, whose energy first decreases and then stabilizes at a rather constant value.

A different behavior is observed at the increment of the distance between QD and DQR. The energies of the lowest three states increase and then remain constant, but the energies of the upper states decrease, first rapidly and then more slowly.

In the simulation of the absorption, we considered only the transitions starting from the ground state. The spectra were analyzed in relation with the oscillator strength of the transitions involved. Because the ground state and the excited states are differently confined in the semiconductor structure, the spectra consist of either a single peak of low energy or of two peaks well separated in energy, some of them of rather high intensity. The increment of quantum dot height gives a good control of the blue-shift of all absorption peaks. A proper increasing of the dimensions of each of the quantum rings tunes the red-shift of all peaks. The increment of the distance between QD and DQR induces the blue-shift of the low energy peak and the red-shift of the high energy peak.

The structure of the quantum dot–double quantum ring shows an increased versatility compared to the quantum dot–single ring, not only because of the increased number of control parameters that more sensitively adjust the electronic and optical properties. The presence of a second ring facilitates a strong coupling in QDDR structure, generating new single states and more anti-crossings in energy, changing accordingly the localization probability of the electrons, and consequently the transport properties.

Our results show that this type of configuration allows a selective tuning of the geometry to confine the electron density within the dot or within each individual ring, a property which is particularly important to quantum transport and quantum information processing.

## Data Availability

The datasets generated and/or analyzed during the current study are available from the corresponding author on reasonable request.
